# Bio-boosting transplants: a systematic review on biopolymers in vascular composite allotransplantation

**DOI:** 10.3389/fimmu.2025.1645261

**Published:** 2026-01-19

**Authors:** Leonard Knoedler, Tobias Niederegger, Thomas Schaschinger, Jakob Fenske, Varun P. A. Murugan, Samuel Knoedler, Max Heiland, Adriana C. Panayi, Gabriel Hundeshagen, Alexandre G. Lellouch

**Affiliations:** 1 Department of Oral and Maxillofacial Surgery, Charité–Universitätsmedizin Berlin, Corporate Member of Freie Universität Berlin and Humboldt-Universität zu Berlin, Berlin, Germany; 2 Division of Plastic and Reconstructive Surgery, Cedars-Sinai Medical Center, Los Angeles, CA, United States; 3 University of Heidelberg, Medical Faculty Heidelberg, Heidelberg, Germany; 4 Department of Chemical Engineering and Materials Science, Amrita School of Engineering, Amrita Vishwa Vidyapeetham, Coimbatore, India; 5 Division of Plastic Surgery, Department of Surgery, Yale School of Medicine, New Haven, CT, United States; 6 Department of Hand, Plastic and Reconstructive Surgery, Burn Center, BG Trauma Center Ludwigshafen, University of Heidelberg, Ludwigshafen, Germany; 7 Innovative Therapies in Haemostasis, INSERM UMR-S 1140, University of Paris, Paris, France; 8 Vascularized Composite Allotransplantation Laboratory, Massachusetts General Hospital, Harvard Medical School, Boston, MA, United States; 9 AP-HP, Hôpital Européen Georges Pompidou, Hematology Department, Paris, France

**Keywords:** biopolymers, immunology, polymers, transplantation, vascularized composite allotransplantation, VCA

## Abstract

**Background:**

Vascularized composite allotransplantation (VCA) joins skin, muscle, bone, nerve, and vessels into a single graft that is both highly immunogenic and mechanically complex. Biopolymers, natural or synthetic, can provide structural scaffolding, localized drug release, and immune modulation. Although widely explored in solid-organ transplantation, their utility in VCA is poorly defined. We therefore conducted a systematic review to consolidate current evidence and map translational priorities.

**Methods:**

Adhering to PRISMA 2020 and registered in PROSPERO (CRD420251039845), we searched PubMed, Web of Science, EMBASE, Cochrane Library, and Google Scholar through April 2025. Original studies evaluating biopolymers in any VCA-relevant setting (*in vitro*, animal, or clinical) were eligible. Clinical quality was judged with the Newcastle-Ottawa Scale and pre-clinical studies with the SYRCLE tool. Given methodological heterogeneity, findings were narratively synthesized.

**Results:**

Eleven studies published between 2014 and 2024 fulfilled inclusion criteria. Collectively, they demonstrate that biopolymers, ranging from decellularized limb and auricular scaffolds to collagen-hydroxyapatite or polycaprolactone bone substitutes, hyaluronic-acid–functionalized vascular grafts, chitosan- or alginate-based drug-eluting coatings, and extracellular-matrix (ECM) sheets delivering cytotoxic T-lymphocyte-associated protein 4-immunoglobulin (CTLA4-Ig) with or without rapamycin, consistently enhance vascularization, support multi-tissue regeneration, and preserve mechanical integrity across diverse VCA models. Immunologically, polymer platforms bias host responses toward tolerance: in a murine hind-limb model, ECM combined with CTLA4-Ig and rapamycin extended graft survival to 72 days while promoting pro-regenerative macrophage polarization. Drug-delivery applications also proved effective; calcium-alginate coatings prolonged vancomycin release for up to 50 days *in vitro*, highlighting the potential for infection control during graft integration. Notwithstanding these benefits, chitosan scaffolds displayed inadequate load-bearing capacity, and heterogeneity in species, graft types, follow-up intervals, and outcome metrics limited direct comparison and impeded meta-analysis.

**Conclusion:**

Biopolymers emerge as potential adaptable platforms that merge mechanical support with finely tuned immune regulation in VCA. Successful translation will depend on tissue-specific material optimization, standardized immunological endpoints, and multicenter studies that replicate clinical complexity. Drawing on lessons from solid-organ transplantation and fostering collaboration among immunologists, biomaterial scientists, and surgeons will be pivotal to moving these technologies from bench to bedside in VCA.

## Graphical Abstract

**Figure:**
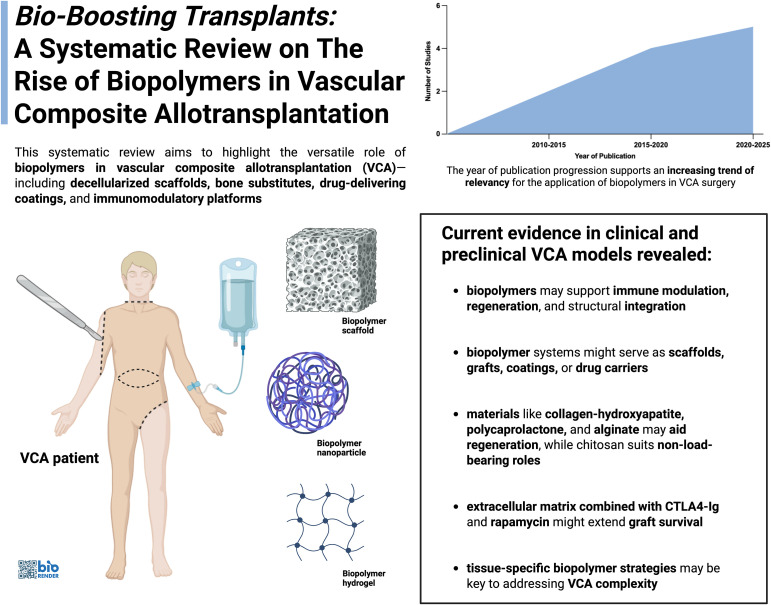

## 1 Introduction

In vascularized composite allotransplantation (VCA), the integration and long-term viability of complex, multi-tissue grafts is impacted by a range of immunological, functional, and regenerative challenges (1–5). While surgical advancements and immunosuppressive protocols have improved graft survival, issues related to tissue regeneration, biomimicry, and host-graft interface healing continue to limit broader clinical adoption and long-term success (1, 3, 4, 6).

Biopolymers, naturally-derived or bioengineered polymeric materials, have emerged as promising adjuncts in VCA, offering diverse applications across immunomodulation, tissue scaffolding, wound healing, and drug delivery (7, 8). Their tunable physicochemical properties, biodegradability, and potential for bioactive modification position them as ideal candidates to support composite tissue integration and functional recovery. In particular, the use of biopolymers as immunosuppressive carriers, nerve conduits, vascular scaffolds, and dermal substitutes has shown encouraging results in preclinical models and select clinical applications (9–14).

Furthermore, insights from solid organ transplantation (SOT), where biopolymers have successfully been used for targeted immunosuppression and bioscaffold support in heart, liver, kidney, pancreas and small intestine transplantation, may help inform and accelerate their application in the more complex, multi-tissue context of VCA (15, 16). Despite this promise, the role of biopolymers in VCA remains fragmented across a heterogeneous body of literature, with limited clinical translation and few standardized approaches (17). Key challenges include the optimization of polymer composition for specific tissue types, ensuring biocompatibility with allograft components, and integrating controlled-release systems within complex vascularized constructs. Furthermore, regulatory barriers and variability in transplant protocols have hindered the systematic evaluation of biopolymer-based interventions (18–20).

Given these considerations, a comprehensive synthesis of current evidence is needed to evaluate the roles, mechanisms, and therapeutic potential of biopolymers in VCA. Therefore, this systematic review aims to consolidate existing research, assess reported outcomes, and highlight opportunities for translational innovation, ultimately informing future clinical strategies and bioengineered solutions in composite tissue transplantation. To ensure a comprehensive overview, this includes not only the direct application of biopolymers but also the assessment of emerging approaches such as ex vivo modifications, *in vitro* and *in vivo* bio-boosting of allotransplants, and deep structural modifications of grafts, including decellularization and recellularization techniques designed to enhance graft integration, immune compatibility, and long-term function.

## 2 Methods

This systematic review adhered to the Preferred Reporting Items for Systematic Reviews and Meta-Analyses (PRISMA) 2020 guidelines. Due to the anticipated variability in study methodologies and reported outcomes, a narrative synthesis was employed in place of a meta-analysis. The review protocol was prospectively registered in the PROSPERO database (ID: CRD420251039845).

*Systematic Search*

A systematic literature search was carried out across PubMed/MEDLINE, EMBASE, the Cochrane Library, Web of Science, and Google Scholar (first 25 pages) for all studies published up to April 20^th^, 2025. The search strategy centered on two main conceptual domains, combined with the Boolean operator “AND”: (i) “vascularized composite allotransplantation (VCA)” and (ii) “biopolymers.” Within each domain, relevant synonyms and MeSH terms were utilized to maximize search sensitivity and comprehensiveness. The full search strings for each database are presented in **Supplementary Digital Content 1**. Additionally, the reference lists of all included articles were reviewed to capture any further eligible studies.

Studies were included if they explored the use, mechanism, or therapeutic impact of biopolymers in the context of VCA, employing clinical data, preclinical animal models, or *in vitro* systems relevant to composite tissue transplantation. All original, peer-reviewed article types—regardless of study design—were considered, provided they were published in English and available as full-text articles. Exclusion criteria comprised non-peer-reviewed literature, articles lacking original data (such as reviews or editorials), or studies evaluating biopolymers outside the scope of VCA or its relevant composite tissue elements.

**Figure 1:**
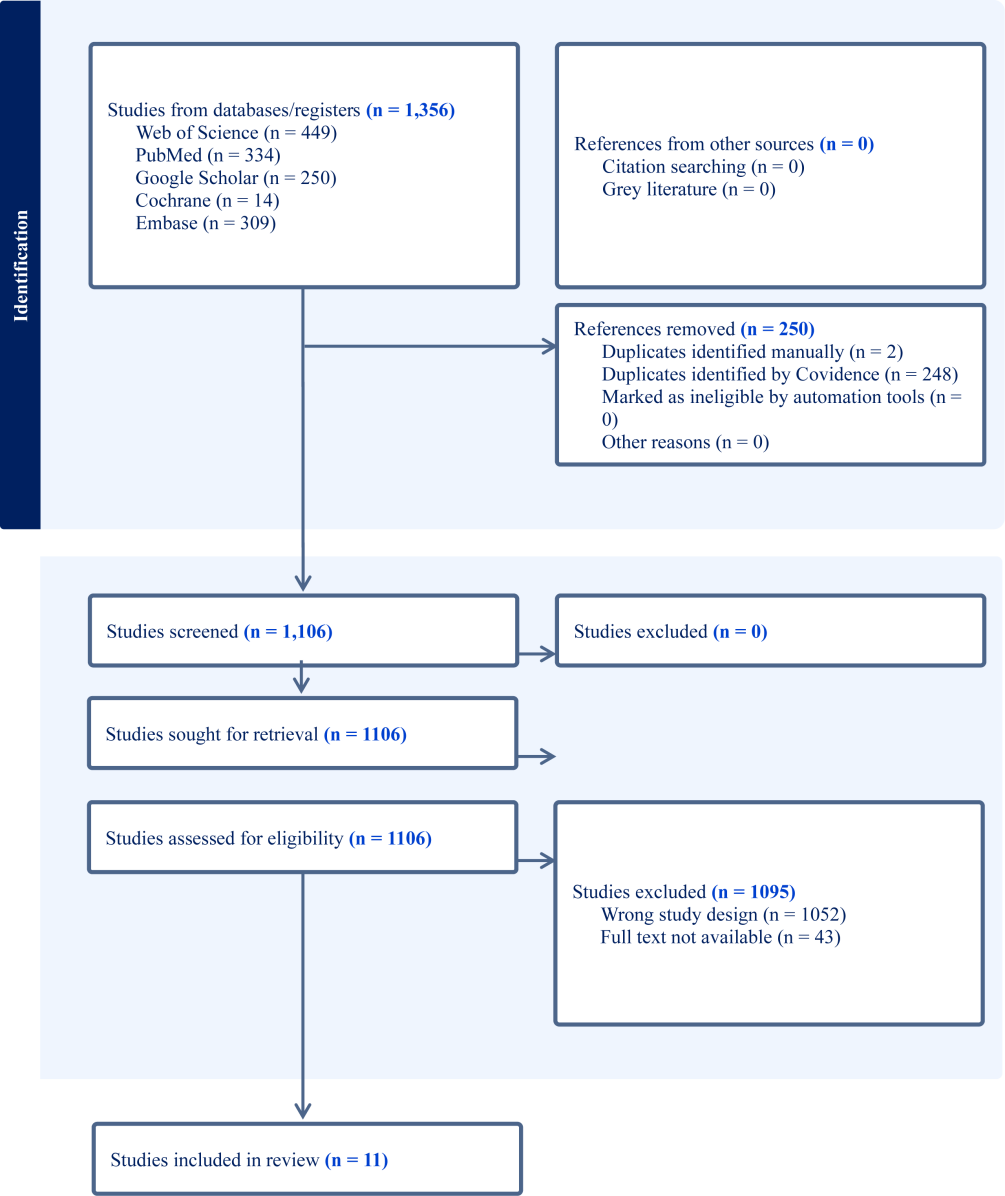
PRISMA 2020 flowchart highlighting the full study selection process.

Title and abstract screening were independently conducted by three reviewers (T.N., T.S., V.M.), followed by full-text assessment for eligibility. Any disagreements were resolved through consensus discussion with a senior reviewer (L.K.). The study selection process is illustrated in the PRISMA 2020 flow diagram provided in **Figure 1**.

*Quality assessment*

The methodological quality of included studies was appraised using validated tools based on study design. Clinical studies were assessed using the Newcastle-Ottawa Scale (NOS), which evaluates methodological rigor across three domains: (i) selection of study groups, (ii) comparability of cohorts, and (iii) ascertainment of outcomes. A maximum of nine stars indicates the highest quality. The mean and standard deviation (SD) of studies included were calculated and reported in the results section (21). For preclinical animal studies, the SYRCLE Risk of Bias tool was employed to evaluate internal validity across key domains such as selection, performance, detection, and reporting bias (22).

In addition, the Oxford Centre for Evidence-Based Medicine (OCEBM) Levels of Evidence (LOE) framework was applied to stratify the strength of evidence (23). Randomized controlled trials and systematic reviews were classified as Level I, while observational studies and preclinical data were ranked according to their design and translational applicability. Detailed results of the quality assessments are provided in **Supplementary Digital Content 2**–**4**.

*Data extraction*

Data extraction was performed through a blinded dual-review process to ensure accuracy and minimize bias. From each included study, the following parameters were systematically collected: Digital Object Identifier (24), study title, first author, year of publication, study type, sample size, recipient age and sex, donor age and sex, duration of follow-up, underlying cause of transplantation, type of VCA, study intervention, objective of intervention, biomaterial used, animal model (if applicable), comparison group (if applicable), and overall study outcome.

## 3 Results

The systematic search initially identified n=1,356 studies. Following application of predefined inclusion and exclusion criteria, a total of n=11 (0.8%) studies met eligibility for final inclusion. Year of publication ranged from 2014 to 2024. The mean (SD) NOS-score was 7.4 (1.5), indicating moderate methodological rigor. The LOE ranged from Foundational Evidence to Level 1b.

Study designs encompassed randomized clinical trials, prospective clinical investigations, as well as preclinical *in vivo* and *in vitro* experimental models, reflecting a multidisciplinary exploration of biopolymer applications in VCA-related fields. Animal models included Wistar rats, Sprague Dawley rats, baboons, and sheep. Transplant types ranged from bone and soft tissue grafts to composite limbs and vascular grafts. Full insights on study designs and cohorts are shown in **Table 1**. A clear definition and classification of biopolymers in this context are provided in **Table 2**.

**Table 1:** Study designs and study cohorts of included articles.

DOI	Author	Title	Year of publication	Study type	Study category	Study population	VCA type/tissue transplant type
DOI: 10.1155/2014/459867	Hornyák et al.	Increased Release Time of Antibiotics from Bone Allografts through a Novel Biodegradable Coating	2014	Experimental Study	Controlled Drug release	Human bone	Bone Allografts (lyophilized femoral head)
DOI: 10.1016/j.biomaterials.2015.04.051	Jank et al.	Engineered Composite Tissue as a Bioartificial Limb Graft	2015	*In vitro* and *in vivo*	Regenerative Medicine	Sprague Dawley rats and non-human primates (baboons)	Forearm (Composite Tissue)
DOI: 10.1002/JPER.17-0466	Clark et al.	Advanced platelet-rich fibrin and freeze-dried bone allograft for ridge preservation: A randomized controlled clinical trial	2018	Randomized controlled clinical trial	Ridge preservation in dental implantology	Human patients (n=40 completed the study)	Autologous (A-PRF), Allogeneic (FDBA) bone
DOI: 10.1155/2018/9430989	La Monaca et al.	Comparative Histological and Histomorphometric Results of Six Biomaterials Used in Two-Stage Maxillary Sinus Augmentation Model after 6-Month Healing	2018	Clinical Study	Bone Regeneration/Implant Dentistry	Human (n=6 patients, 3 males, 3 females, aged 50–72 years)	Maxillary Sinus Augmentation
DOI: 10.1016/j.actbio.2018.04.009	Duisit et al.	Perfusion-decellularization of human ear grafts enables ECM-based scaffolds for auricular vascularized composite tissue engineering	2018	*In vitro* and *in vivo*	Decellularization	n=16 adult Wistar rats (female)	Human ear grafts
DOI: 10.1016/j.apsusc.2020.147196	Kudryavtseva et al.	Magnetron plasma mediated immobilization of hyaluronic acid for the development of functional double-sided biodegradable vascular graft	2020	*In vitro* and preliminary *in vivo* evaluation	Vascular Graft Development	Human mesenchymal stem cells	Small diameter vascular graft
DOI: 10.1111/clr.13911	Abellán et.al	Ridge preservation in molar sites comparing xenograft versus mineralized freeze-dried bone allograft: A randomized clinical trial	2021	Randomized clinical trial	Alveolar ridge preservation	Humans (Male: n=8 (38.1%), Female: n=13 (61.9%))	Xenograft (DBBM) and mineralized freeze-dried bone allograft (FDBA)
DOI: 10.1111/cid.13124	Gallo et al.	Comparative analysis of two biomaterials mixed with autogenous bone graft for vertical ridge augmentation: A histomorphometric study in humans	2022	Prospective clinical trial	Vertical ridge augmentation (VRA), Guided bone regeneration (GBR)	n=16 partially edentulous human patients	Autogenous bone mixed with allograft (FDBA) or xenograft (bovine)
DOI: 10.17219/dmp/166229	Naidu et al.	Comparative study of demineralized freeze-dried bone allograft and its combination with platelet rich fibrin in the treatment of intrabony defects: A randomized clinical trial	2023	Randomized clinical trial	Periodontal regeneration	Human (n=12 systemically healthy patients with intrabony defects)	Allograft (DFDBA)
DOI: 10.1039/d2bm01845d	Sommerfeld et al.	Biomaterials-based immunomodulation enhances survival of murine vascularized composite allografts	2023	*In vivo* and *in vitro*	Immunomodulation using biomaterials	Murine model (BALB/c to C57BL/6 mice)	Orthotopic hindlimb vascularized composite allograft
DOI: 10.1007/s00223-024-01309-x	Knudsen et al.	Long-Term Natural Hydroxyapatite and Synthetic Collagen Hydroxyapatite Enhance Bone Regeneration and Implant Fixation Similar to Allograft in a Sheep Model of Implant Integration	2024	*In vivo* animal study	Bone regeneration, orthopedic biomaterials, implant fixation	n=14 skeletally mature female Texel/Gotland sheep (age: 6 ± 1 years, weight: 73.4 ± 7.6 kg).	Sheep femurs

DOI, Digital Object Identifier; ECM, Extracellular Matrix; A-PRF, Advanced Platelet-Rich Fibrin; FDBA, Freeze-Dried Bone Allograft; DFDBA, Demineralized Freeze-Dried Bone Allograft; DBBM, Deproteinized Bovine Bone Mineral; COL/HA, Collagen-Hydroxyapatite; nHA, Natural Hydroxyapatite; CS/HA, Chitosan-Hydroxyapatite; VCA, Vascularized Composite Allotransplantation; VMT, Vital Mineralized Tissue; GBR, Guided Bone Regeneration.

**Table 2:** Biopolymer categories, definitions, subtypes, example materials, and applications in vascularized composite allotransplantation.

Category	Definition	Subtypes	Example materials	Applications in VCA
Biopolymers (general)	Naturally-derived or synthetic polymers that are biocompatible, biodegradable, and capable of supporting structural or biological functions in transplanted tissues	Natural and synthetic	Chitosan, alginate, collagen, hyaluronic acid, polycaprolactone (PCL), hydroxyapatite (HA)	Scaffolding, drug delivery, immunomodulation, vascular and neural integration
Natural biopolymers	Derived from biological sources; often mimic native ecm and support cell adhesion and growth	Polysaccharides, proteins	Chitosan, alginate, collagen, gelatin, hyaluronic acid	Drug-eluting coatings (e.g., vancomycin), dermal scaffolds, immunomodulatory matrices
Synthetic biopolymers	Engineered polymers with controllable physical and degradation properties	Polyesters, polyanhydrides	Polycaprolactone (PCL), polylactid-co-glycolid (PLGA), polylactic acid (PLA)	Vascular grafts, load-bearing bone substitutes, nerve conduits
Composite biopolymers	Blends of natural and synthetic materials to enhance mechanical and biological performance	Hybrid constructs	Collagen-HA, chitosan-HA, ECM-derived + immunosuppressants (e.g., CTLA4-Ig, rapamycin)	Bone grafts, immunosuppressive matrices, composite tissue regeneration
Decellularized ECM-based biopolymers	Native tissues processed to remove cellular content, preserving structural proteins and microarchitecture	ECM scaffolds	Decellularized forearms, human auricular grafts, porcine urinary bladder matrix (e.g. MatriStem)	Scaffold for limb/facial grafts, immune tolerance platforms
Drug-delivery biopolymer platforms	Biopolymers designed to release therapeutic agents locally and in a sustained manner	Hydrogels, coatings	Calcium-alginate, chitosan, polylactid-co-glycolid (PLGA) microspheres, ECM sheets	Local immunosuppression, infection prevention, controlled antibiotic delivery

ECM, Extracellular Matrix; PCL, Polycaprolactone; HA, Hydroxyapatite; PLGA, Polylactid-co-glycolid; PLA, Polylactic Acid; HA, Hyaluronic Acid; CTLA4-Ig, Cytotoxic T-Lymphocyte Antigen 4 Immunoglobulin; VCA, Vascularized Composite Allotransplantation.

### 3.1 Direct VCA Applications: Biopolymer Scaffolds for Composite Tissue Engineering

Jank et al. employed perfusion-decellularization and recellularization of rat and primate forearms, preserving extracellular matrix (ECM) architecture and enabling repopulation with myoblasts, fibroblasts, and endothelial cells, ultimately producing contractile, muscle-like constructs. This work established a foundational framework for patient-specific, immunosuppression-free VCA graft engineering (25). Similarly, Duisit et al. applied perfusion-decellularization to human auricular grafts, achieving a 97% reduction in DNA while retaining ECM organization and vascular microarchitecture. When implanted in Wistar rats, these acellular scaffolds demonstrated good biocompatibility and minimal immune response, supporting their relevance for facial composite reconstruction (26). Collectively, these studies highlight the versatility of decellularized biopolymer scaffolds for limb and facial VCA, demonstrating preserved architecture, biocompatibility, and the potential for immunologically favorable graft preparation. However, studies did not provide sufficient safety analysis, resulting in a limited translational readiness.

### 3.2 Bone regeneration evidence: Biopolymer-enhanced osseous grafts

Multiple studies evaluated biopolymer-augmented bone substitutes relevant to VCA scenarios involving maxillofacial or craniofacial reconstruction. Clark et al. conducted a randomized controlled trial comparing four ridge preservation techniques and found that advanced platelet-rich fibrin (PRF), alone or combined with freeze-dried bone allograft (FDBA), significantly minimized ridge height loss and promoted vital bone formation, up to 46% compared to 29% with FDBA alone, highlighting its regenerative potential (27). Abellán et al. found that both deproteinized bovine bone mineral (DBBM) and FDBA provided comparable outcomes in molar ridge preservation, with similar vital bone formation and dimensional stability. Thicker buccal bone plates reduced remodeling, and most cases required only minor sinus lifts, highlighting both grafts as flexible, effective options relevant for bone reconstruction in VCA (28). Gallo et al. extended this line of research to vertical ridge augmentation, showing that both bovine xenografts, covered by a nonabsorbable high-density polytetrafluoroethylene (d-PTFE) membrane, and FDBA, when combined with autogenous bone chips, supported high mineralized tissue formation and complete implant survival (29). Preclinical work by Knudsen et al. reinforced these trends, revealing that natural hydroxyapatite (nHA) and collagen-hydroxyapatite (COL/HA) composites were as effective as traditional allografts in promoting bone regeneration and implant fixation in a distal femoral condyle bone graft sheep model. In contrast, chitosan-hydroxyapatite (CS/HA) composites showed poor osteoconductivity and mechanical performance, suggesting that while chitosan holds promise, its use as a load-bearing scaffold may require further optimization (30). La Monaca et al. conducted a comparative histomorphometric analysis of six bone graft materials used in maxillary sinus augmentation and found that FDBA achieved the highest percentage of new bone formation (32%), with no adverse events or implant failures, confirming its strong biocompatibility and efficacy for vertical bone regeneration (31).

In contrast, Naidu et al. evaluated the use of PRF in combination with demineralized FDBA (DFDBA) for periodontal regeneration and observed no significant improvement over DFDBA alone, highlighting that while FDBA consistently supports bone formation, pairing it with certain adjuncts like PRF may not yield synergistic effects (32).

### 3.3 Vascular engineering: Polymer-based grafts supporting vascular integration

For VCA graft survival, early and stable vascularization is essential. Kudryavtseva et al. engineered small-diameter vascular grafts from electrospun polycaprolactone (PCL), enhanced via magnetron plasma treatment and hyaluronic acid (HA) functionalization. This dual modification produced a hydrophilic outer surface that promoted robust cell adhesion and a hydrophobic inner surface that maintained mechanical stability. The study utilized human adipose-derived mesenchymal stem cells (CD19^-^CD34^-^CD45^-^CD73^+^CD90^+^CD105^+^ MSCs), which adhered efficiently to the HA-coated surface and exhibited improved morphology, intercellular connectivity, and syncytial formation, which are key for endothelialization. The authors highlighted that these properties are particularly relevant for VCA, where long-term graft survival hinges on rapid and stable vascular integration. The dual-modified PCL grafts thus offer a promising, bioactive scaffold platform to support stem cell-mediated vascular repair in complex composite tissue transplants, key requirements for successful VCA graft perfusion (33). Overall, these results position engineered PCL-based vascular scaffolds as potential future candidate adjuncts for restoring or enhancing microvascular networks in complex composite tissue transplants, if safety concerns are met.

### 3.4 Immune and drug-delivery modulation: Biopolymers as immunoregulatory platforms

Several studies investigated the immunomodulatory potential of biopolymer systems in transplantation contexts relevant to VCA. Hornyák et al., investigated chitosan and calcium alginate coatings on composite grafts for localized antibiotic delivery. While chitosan coatings did not significantly prolong drug release, calcium alginate coatings extended vancomycin release up to 50 days, underscoring chitosan’s limitations in structural applications but supporting its use in antimicrobial delivery during graft integration (34). Sommerfeld et al. demonstrated that local application of a decellularized porcine urinary bladder matrix (MatriStem), in combination with cytotoxic T-lymphocyte-associated protein 4-immunoglobulin (CTLA4-Ig) and rapamycin, significantly prolonged graft survival in a murine hindlimb VCA model, extending median survival time to 72.5 days and promoting a pro-regenerative immune environment (11).

### 3.5 Summary and Safety

Organizing the available evidence across composite graft scaffolds, bone regeneration, vascular engineering, and immune/drug delivery systems highlights the wide-ranging applications of biopolymers in VCA. These materials demonstrate adaptability across tissues, relevance for functional integration, and promise for future translational pathways. However, consistent reporting on safety considerations, such as degradation byproducts, foreign-body responses, and long-term biocompatibility, remains limited across studies, raising concerns about unrecognized failure modes and potential selection bias toward positive outcomes. Full data are summarized in **Table 3**.

**Table 3:** Overview of biopolymer-enhanced constructs in VCA-targeted research.

DOI	Author	Title	Year of publication	Intervention	Objective of intervention	Biomaterials used	Additional drugs	Comparison groups	Maximum follow up duration	Outcome
DOI: 10.1155/2014/459867	Hornyák et al.	Increased Release Time of Antibiotics from Bone Allografts through a Novel Biodegradable Coating	2014	Biodegradable antibiotic coated implant for a sustained release of antibiotics	Prevention of bacterial infection	Chitosan (Chi) and Sodium Alginate (Na-Alg)	Amoxicillin, ciprofloxacin, or vancomycin	Antibiotic coated grafts (soaked vs. saturated methods), antibiotic + chitosan coated grafts, antibiotic + alginate coated grafts	50 days	Simple freeze drying resulted in short-term antibiotic release (up to 48 hours); Chitosan coating did not significantly prolong release; Calcium alginate coating enabled sustained release: amoxicillin: 8 days, ciprofloxacin: 28 days, vancomycin: 50 days
DOI: 10.1016/j.biomaterials.2015.04.051	Jank et al.	Engineered Composite Tissue as a Bioartificial Limb Graft	2015	Perfusion decellularization and recellularization of rat and primate forearms, followed by transplantation	To create a bioartificial limb graft using decellularized scaffolds repopulated with patient derived cells, avoiding immunosuppression	Acellular extracellular matrix (ECM)	N/A	Native vs. decellularized forearms. Recellularized grafts vs. native tissue	21days	Successful decellularization with preserved ECM architecture and mechanical properties; Recellularization with myoblasts, endothelial cells, and fibroblasts led to functional muscle like tissue formation
DOI: 10.1002/JPER.17-0466	Clark et al.	Advanced platelet-rich fibrin and freeze-dried bone allograft for ridge preservation: A randomized controlled clinical trial	2018	Four ridge preservation approaches: A-PRF alone, A-PRF+FDBA, FDBA alone, or blood clot (control)	To evaluate the efficacy of A-PRF alone or with FDBA in improving vital bone formation and alveolar dimensional stability post extraction	Advanced platelet rich fibrin (A-PRF), freeze dried bone allograft (FDBA), collagen dressing	N/A	A-PRF alone, A-PRF+FDB, FDBA alone, Blood clot (control)	15 weeks	Ridge Preservation: A-PRF and A-PRF+FDBA showed significantly less ridge height reduction compared to blood clot; Vital Bone Formation: A-PRF alone had the highest vital bone formation (46% ± 18%), significantly greater than FDBA (29% ± 14%); Bone Mineral Density: FDBA had higher bone mineral density than blood clot, but A-PRF performed comparably to FDBA
DOI: 10.1155/2018/9430989	La Monaca et al.	Comparative Histological and Histomorphometric Results of Six Biomaterials Used in Two-Stage Maxillary Sinus Augmentation Model after 6-Month Healing	2018	Two stage sinus lift augmentation with delayed implant placement using six different biomaterials	To evaluate the histological and histomorphometric performance of six bone substitute materials in maxillary sinus augmentation	Mineralized solvent dehydrated bone allograft (MCBA), Freeze-dried mineralized bone allograft (FDBA), anorganic bovine bone (ABB), equine-derived bone (EB), synthetic micro-macroporous biphasic calcium, phosphate (HA-β-TCP 30/70), bioapatite-collagen (BC)	Preoperative antibiotic: amoxicillin 875 mg + clavulanic acid 125 mg AND analgesic: ketoprofene 200 mg; antiseptic rinse: chlorhexidine digluconate 0.2%	Six biomaterials (MCBA, FDBA, ABB, EB, HA-β-TCP 30/70, BC) compared histologically and histomorphometrically	12–14 months	All biomaterials showed good biocompatibility and osteoconductive properties; FDBA had the highest percentage of newly formed bone (32.1%); No postoperative complications or implant failures occurred
DOI: 10.1016/j.actbio.2018.04.009	Duisit et al.	Perfusion-decellularization of human ear grafts enables ECM-based scaffolds for auricular vascularized composite tissue engineering	2018	Perfusion-decellularization of human ear grafts using SDS/polar solvent protocol	To create a biocompatible, vascularized, and complex auricular scaffold for tissue engineering applications	Decellularized extracellular matrix (ECM)	N/A	Native human ear grafts vs. decellularized human ear scaffolds	60 days	Successful decellularization with 97.3% DNA reduction; Biocompatibility confirmed *in vivo* with limited immune response
DOI: 10.1016/j.apsusc.2020.147196	Kudryavtseva et al.	Magnetron plasma mediated immobilization of hyaluronic acid for the development of functional double-sided biodegradable vascular graft	2020	Two-stage processing of electrospun PCL grafts - (1) DC magnetron plasma treatment, (2) hyaluronic acid (HA) immobilization	To create a double-sided vascular graft with a hydrophilic outer surface (for cell adhesion) and hydrophobic inner surface (for mechanical integrity)	Polycaprolactone (PCL)	N/A	Untreated PCL grafts, plasma treated PCL grafts (varying power levels: 20W, 45W, 75W, 105W, 135W), plasma-treated + HA immobilized PCL grafts	72 hours	Plasma treatment improved hydrophilicity and cell adhesion; HA immobilization prevented hydrophobic recovery and enhanced long-term stability
DOI: 10.1111/clr.13911	Abellán et.al	Ridge preservation in molar sites comparing xenograft versus mineralized freeze-dried bone allograft: A randomized clinical trial	2021	Ridge preservation using DBBM or FDBA with a resorbable collagen membrane	To compare dimensional ridge changes and histological composition after ridge preservation in molar sites using DBBM or FDBA, and to evaluate the influence of bone plate thickness and the need for sinus augmentation	DBBM (Bio-Oss, Geistlich Pharma), FDBA (MinerOss, BioHorizons), resorbable collagen membrane (Mem-lok, BioHorizons)	Antibiotics: amoxicillin or clindamycin (for penicillin-allergic patients), analgesics: ibuprofen or paracetamol, antiseptic rinse: 0.12% chlorhexidine digluconate and 0.05% cetylpyridinium chloride (Perio-Aid)	DBBM group, FDBA group	5 months	Both materials resulted in similar dimensional changes and histomorphometric outcomes; Thicker buccal bone plates exhibited less bone remodeling; No significant differences in vital bone formation between groups; 55% of preserved sites required transcrestal sinus lift, but none needed lateral augmentation
DOI: 10.1111/cid.13124	Gallo et al.	Comparative analysis of two biomaterials mixed with autogenous bone graft for vertical ridge augmentation: A histomorphometric study in humans	2022	Vertical ridge augmentation using autogenous bone chips mixed with either freeze dried bone allograft (FDBA) or bovine xenograft, covered by a nonabsorbable high-density polytetrafluoroethylene (d-PTFE) membrane	To assess vital mineralized tissue formation in VRA procedures using autogenous bone chips mixed with either an allograft or a xenograft	Freeze dried bone allograft (FDBA) (MinerOs; Biohorizons), bovine xenograft (Zcore; Osteogenics Biomedical), autogenous bone chips, titanium-reinforced d-PTFE membrane (Cytoplast; Osteogenics Biomedical)	Amoxicillin and clavulanate potassium (antibiotic), nimesulid (anti-inflammatory), 0.12% chlorhexidine solution (antiseptic rinse)	Group A: FDBA + autogenous bone AND Group B: Xenograft + autogenous bone	92 weeks	No significant difference in vital mineralized tissue (VMT) formation between groups (67.64% ± 16.84% for Group A vs. 60.93% ± 18.25% for Group B); Both biomaterials, when mixed with autogenous bone, provided successful vertical ridge augmentation with high percentages of VMT; 100% implant survival rate in both groups
DOI: 10.17219/dmp/166229	Naidu et al.	Comparative study of demineralized freeze-dried bone allograft and its combination with platelet rich fibrin in the treatment of intrabony defects: A randomized clinical trial	2023	DFDBA alone vs. DFDBA combined with PRF	To compare the clinical and radiographic effectiveness of DFDBA with PRF versus DFDBA alone in treating intrabony defects	Demineralized freeze-dried bone allograft (DFDBA), platelet-rich fibrin (PRF)	N/A	Group A: DFDBA alone, Group B: DFDBA + PRF	9 months	Both groups showed significant improvements in clinical and radiographic parameters, but no statistically significant differences were observed; PRF did not provide additional benefits over DFDBA alone
DOI: 10.1039/d2bm01845d	Sommerfeld et al.	Biomaterials-based immunomodulation enhances survival of murine vascularized composite allografts	2023	Local ECM application with systemic CTLA4-Ig ± rapamycin	Enhance allograft survival and modulate immune response	Decellularized porcine urinary bladder matrix (MatriStem)	CTLA4-Ig, Rapamycin	Untreated, ECM only, CTLA4-Ig only, CTLA4-Ig + ECM, CTLA4-Ig + rapamycin, CTLA4-Ig + rapamycin + ECM	80 days	ECM alone ineffective; ECM + CTLA4-Ig increased MST to 24.5 days; ECM + CTLA4-Ig + rapamycin increased MST to 72.5 days with pro-regenerative immune shift
DOI: 10.1007/s00223-024-01309-x	Knudsen et al.	Long-Term Natural Hydroxyapatite and Synthetic Collagen Hydroxyapatite Enhance Bone Regeneration and Implant Fixation Similar to Allograft in a Sheep Model of Implant Integration	2024	Circumferential gaps filled with nHA (natural hydroxyapatite), COL/HA (collagen-hydroxyapatite), CS/HA (chitosan-hydroxyapatite), or allograft (control)	To evaluate long term (14 & 24 weeks) efficacy of natural/synthetic HA composites in bone regeneration and implant fixation compared to allograft	nHA (natural HA from oyster shells, pore size: 100–300 µm), COL/HA (bovine type I collagen + HA, ratio 12:1, pore size: 100–350 µm), CS/HA (chitosan + HA, ratio 4:20, irregular porosity), allograft (control, morselized sheep bone, 0.5–1.5 mm particles)	Temgesic (buprenorphine, 0.3 mg/mL) for pain. AND Ampivet (ampicillin, 250 mg/mL) for infection prevention	Allograft (control), nHA (natural HA), COL/HA (collagen HA composite), CS/HA (chitosan HA composite)	24 weeks	nHA & COL/HA performed comparably to allograft in bone formation and implant fixation at 24 weeks; COL/HA showed 52% higher bone volume fraction than allograft (non-significant); CS/HA had poor osteoconductivity, minimal degradation, and fibrous tissue formation; Mechanical strength: COL/HA > nHA ≈ allograft > CS/HA

DOI,Digital Object Identifier; A-PRF,Advanced Platelet-Rich Fibrin; COL/HA,Collagen-Hydroxyapatite; CS/HA,Chitosan-Hydroxyapatite; DFDBA,Demineralized Freeze-Dried Bone Allograft; ECM,Extracellular Matrix; CTLA4-Ig, Cytotoxic T-Lymphocyte Antigen 4 Immunoglobulin; FDBA,Freeze-Dried Bone Allograft; DBBM, Deproteinized Bovine Bone Mineral; HA,Hydroxyapatite; HA-β-TCP,Hydroxyapatite–Beta-Tricalcium Phosphate; nHA,Natural Hydroxyapatite; PCL,Polycaprolactone; PRF,Platelet-Rich Fibrin; SDS,Sodium Dodecyl Sulfate; VCA,Vascularized Composite Allotransplantation; VMT,Vital Mineralized Tissue; d-PTFE,Dense Polytetrafluoroethylene.

## 4 Discussion

**Figure 2:**
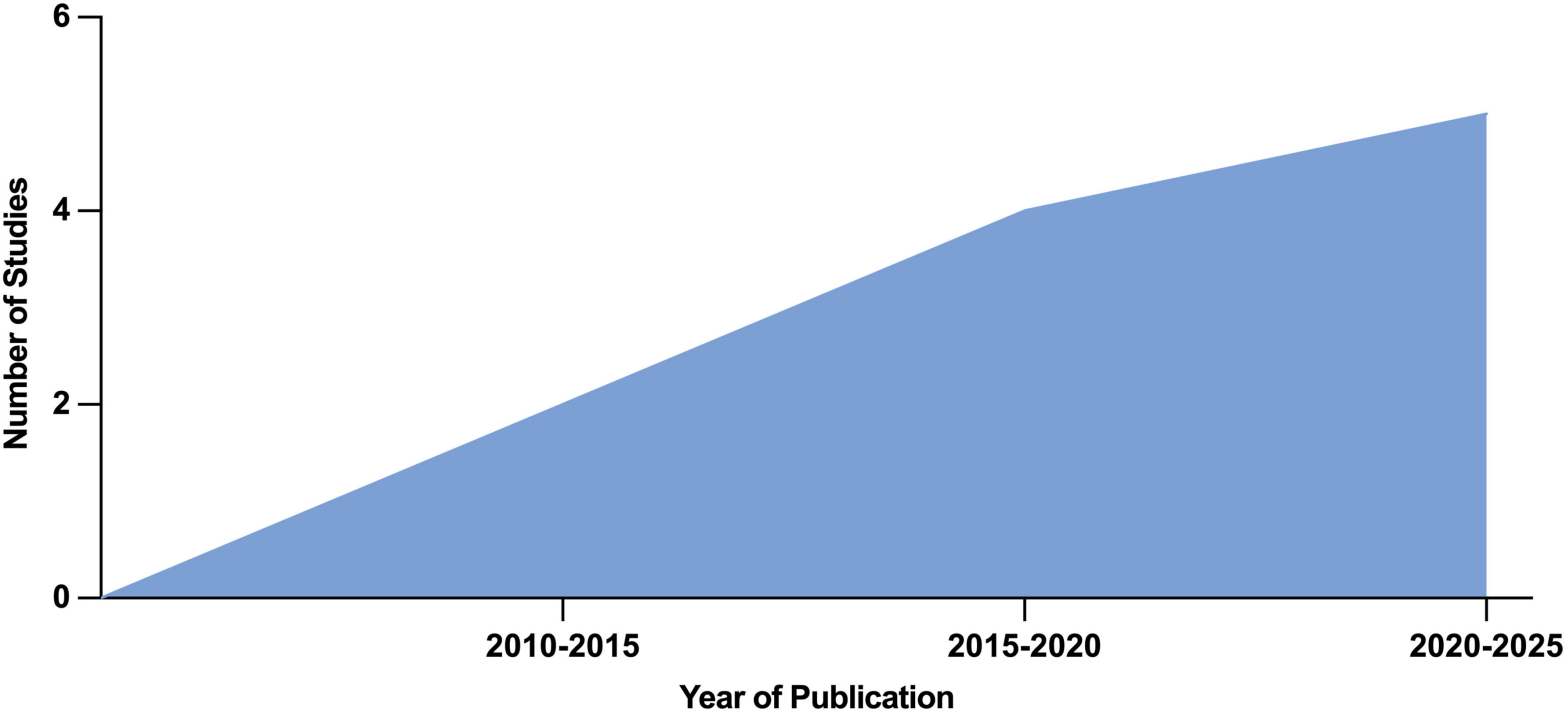
Year of publication progression supporting an increasing trend of relevancy for the application of biopolymers in VCA surgery.

Biopolymers have emerged as promising preclinical adjuncts in VCA due to their biocompatibility, tunable degradation profiles, and potential for bioactive functionalization (**Figure 2**). In this field, where multi-tissue integration and long-term graft viability remain critical challenges, biopolymers offer unique opportunities to enhance outcomes through scaffold support, localized drug delivery, and immunomodulation. Their role spans across structural regeneration, infection prevention, and vascular or neural guidance within complex graft environments. Conversely, in tissue engineering, a major challenge lies in the effective recellularization of the scaffold, which can occur *in vitro* under controlled laboratory conditions or *in vivo* where the human body functions as a natural bioreactor (35). As research advances, biopolymers may serve as key enablers of next-generation, bioengineered solutions that improve both functional recovery and immunologic tolerance in VCA, which, to date, was only achieved *in vivo* through mixed chimerism (7, 8, 36–42).

**Figure 3:**
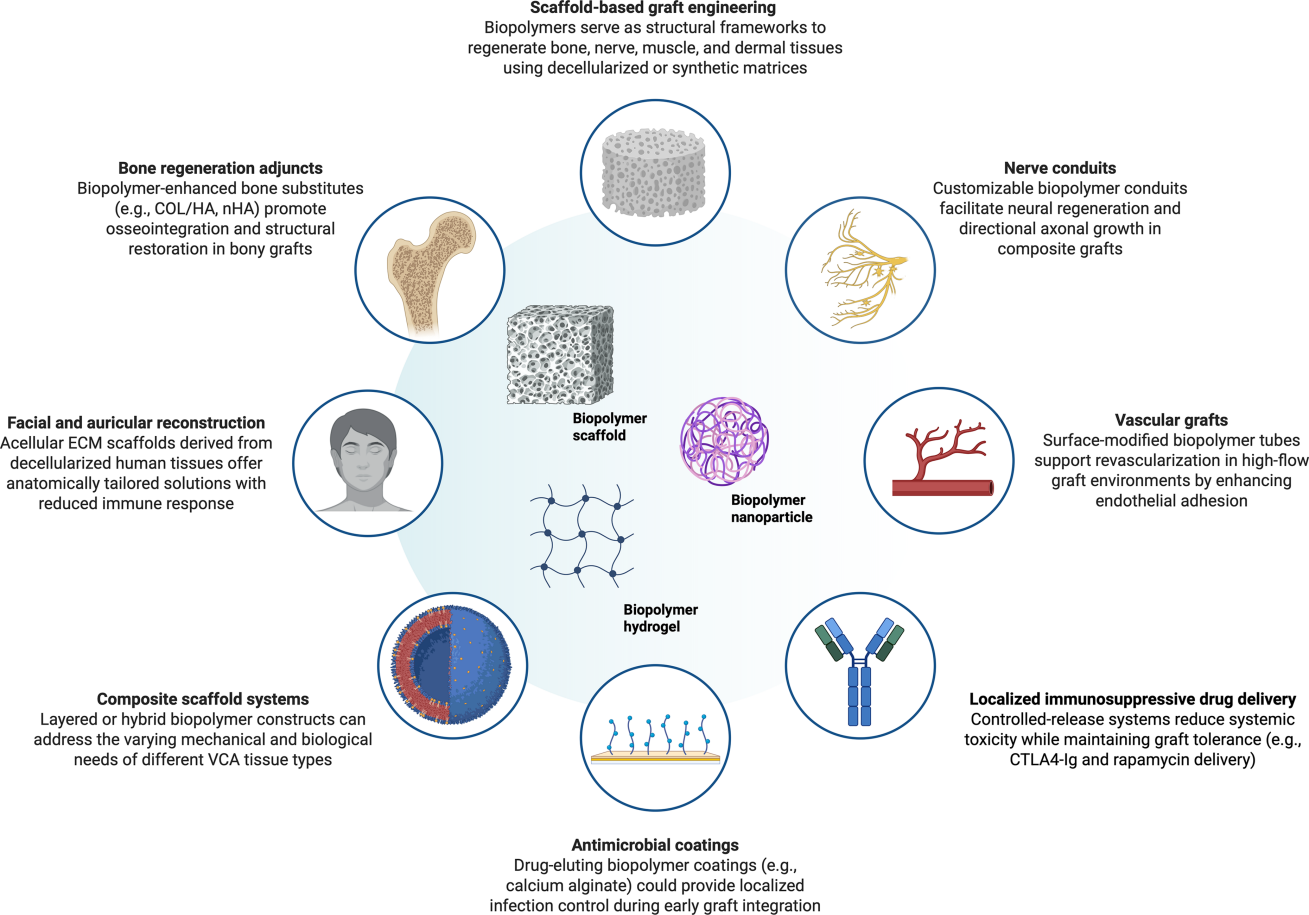
Multifaceted potential applications for biopolymers in VCA surgery. This schematic illustrates the diverse functional roles of biopolymers in VCA, as identified through a systematic review of clinical and preclinical studies. Central biopolymer platforms include scaffolds, hydrogels, and nanoparticles. Surrounding panels highlight key application areas: (1) scaffold-based graft engineering for multi-tissue regeneration; (2) nerve conduits supporting axonal guidance; (3) vascular grafts promoting endothelial adhesion; (4) localized immunosuppressive delivery systems (e.g., CTLA4-Ig, rapamycin); (5) antimicrobial coatings for infection control; (6) composite scaffold systems tailored to multiple tissue types; (7) facial and auricular reconstruction using decellularized ECM; and (8) bone regeneration adjuncts (e.g., COL/HA, nHA). Together, these strategies underscore the potential of biopolymers to enhance immune modulation, integration, and repair in complex transplant environments.

In our study, biopolymers have shown significant versatility in VCA, supporting tissue-specific scaffold development for limb, facial, and vascular grafts through techniques like decellularization and surface modification. While materials such as COL/HA, PCL, and calcium alginate demonstrated strong regenerative or structural properties, others like chitosan faced limitations in load-bearing applications, though remained effective in drug delivery (**Figure 3**).

Current research increasingly highlighted the versatility of biopolymers in modulating immune responses, promoting graft integration, and supporting structural regeneration in transplant surgery (43, 44). Their tunable biodegradability, mechanical properties, and ability to carry bioactive molecules are further advantages (45, 46). In literature, the application of biopolymers in VCA ranges from localized drug delivery systems to structural scaffolds designed to promote cellular adhesion, angiogenesis, or tissue remodeling (47, 48). In this context, biopolymers were evaluated for their efficacy in drug delivery and regenerative capacity in specific tissues (49). Natural and synthetic polymers were incorporated into nerve conduits, vascular scaffolds, dermal replacements, and bone grafts. Literature supported their use in enhancing revascularization, modulating inflammatory responses at the host-graft interface, and creating composite scaffolds that mimicked the structural and biochemical features of native tissues (50, 51). However, biopolymer application faces various challenges. The structural demands of each tissue type meant that a single biopolymer system was rarely suitable across the entire graft (52). Furthermore, skin components oftentimes elicited particularly strong immune responses, making tolerance induction difficult (4, 53–55). Bone regeneration required load-bearing strength, while neural repair called for guidance conduits with bioactive cues, necessitating tailored, often multi-layered or composite polymer systems. The integration of these systems into a single, unified construct that could function *in vivo* remained a significant engineering challenge (56–59). Another issue was the relative scarcity of translational studies. While preclinical models showed encouraging results, clinical implementation lagged due to regulatory constraints, manufacturing scalability, and the need for long-term outcome data (60–63). This gap is particularly problematic because most preclinical studies observe grafts for only weeks to months, whereas VCA recipients require immunosuppression and graft surveillance over decades, creating a disconnect between experimental timelines and real-world clinical needs. In our review, follow-up periods were similarly short, highlighting the challenge of extrapolating short-term biomaterial performance to the lifelong immunosuppression demands faced by VCA patients These challenges are further amplified by the limited number of performed VCA transplants globally, which may have constrained the ability to perform large-scale clinical studies. Additionally, ethical considerations regarding the risk-benefit profile of VCA transplants complicated the evaluation of experimental adjuncts such as biopolymer systems (18, 53, 64). Moreover, biopolymers derived from natural sources often suffer from inherent batch-to-batch variability and structural complexity, making mass production for commercial clinical applications technically and economically challenging, posing a significant hurdle for widespread application (65, 66). Here, triglycerol monostearate (TGM) gels and other injectable hydrogel platforms loaded with tacrolimus emerged as potential alternatives and have shown promise as minimally invasive drug delivery systems due to their thermoresponsive properties, ability to form depots *in situ*, and responsiveness to inflammation-associated enzymatic triggers that enable on-demand drug release. However, challenges such as limited mechanical integrity, potential burst release, and the absence of intrinsic regenerative or structural functions restrict their standalone utility in the multi-tissue environment of VCA (7, 67, 68).

In solid organ transplantation, biopolymers were mainly investigated for targeted immunosuppressive delivery, using microspheres, hydrogels, and nanoparticles to reduce systemic toxicity while enhancing graft-specific tolerance (47). They were also explored for cell encapsulation and early biosensing applications aimed at protecting donor tissue and monitoring graft health (69–72). However, persistent challenges, including incomplete local immunosuppression and the inability to prevent chronic, silent rejection, limited their clinical translation (73–78).

Nevertheless, research continues to explore more sophisticated biopolymer solutions, including stimuli-responsive systems, multi-functional coatings, and bioresorbable materials capable of temporally staged degradation. These innovations promised to enable the fine-tuned orchestration of immune modulation, structural support, and regenerative signaling, all key to advancing outcomes in both SOT and VCA (79–81). While the maturity of biopolymer applications in SOT was more advanced, the multifaceted demands of VCA offered a unique proving ground for next-generation biomaterial technologies.

For patients, biopolymer-enhanced grafts may offer future solutions that promote healing in hand, face, or limb transplants by supporting the regeneration of bone, nerve, and blood vessels while potentially reducing the need for lifelong systemic immunosuppression. For physicians, these platforms provide new potential tools for customizing graft design, ranging from load-bearing bone substitutes to localized drug delivery systems using agents. Although most studies remain in preclinical stages, early findings highlight the translational promise of biopolymers in improving functional outcomes, reducing complications, and expanding therapeutic options in VCA. Lastly, to further contextualize the future translational trajectory of biopolymer technologies in VCA, it is important to define what level of evidence would justify progression toward human application. At present, the available data remain insufficient to support early-phase clinical VCA trials, even at a Phase 0 exploratory or microdosing level, primarily due to short follow-up periods, limited safety reporting, and the absence of robust large-animal efficacy data. Meaningful translation will require well-designed, long-term studies in relevant large-animal VCA models that evaluate degradation behavior, immunologic safety, and functional integration under clinically realistic immunosuppression regimens. From a regulatory perspective, many biopolymer-based VCA adjuncts may be classified as combination products, integrating device, drug, or biologic components, which introduces additional requirements for manufacturing consistency, sterility validation, and dual-pathway oversight through FDA’s CDRH and CBER branches. Only once biopolymers demonstrate reproducible safety, predictable pharmacokinetics, and structural performance in validated large-animal models could regulatory agencies consider first-in-human exploratory use in VCA. As such, translation readiness remains preliminary and contingent upon both scientific and regulatory milestones yet to be achieved.

## 5 Limitations

This systematic review was subject to several limitations that may affect the generalizability of its findings. First, the overall number of studies specifically addressing biopolymer use in VCA remains low, reflecting the nascent and exploratory stage of this field. Many of the included investigations were preclinical in nature, limiting the ability to extrapolate outcomes to human clinical settings. Additionally, heterogeneity in study design, graft type, animal models, and outcome measures hindered direct comparisons and precluded a quantitative meta-analysis. The diversity of polymer types, processing techniques, and application modes further introduced variability that may mask the relative effectiveness of specific biomaterials. Moreover, due to the scarcity of dedicated data, the boundary between biopolymer applications in established VCA, potential translation to VCA, or broader reconstructive contexts often remains blurred, complicating standard classification and comparative assessment. Importantly, reliable reporting on safety considerations, such as degradation products, foreign body responses, and long-term biocompatibility, was largely absent across studies, preventing a rigorous analysis of failure modes and raising the possibility of inadvertent positive-outcome bias, which in turn limits the translational readiness of the current evidence. Publication bias is also a concern, as studies demonstrating negative or inconclusive outcomes may be underrepresented in the literature. Lastly, regulatory, ethical, and logistical barriers specific to VCA constrain the availability of high-quality, large-scale clinical data, underscoring the need for standardized reporting frameworks and multicenter collaboration in future research.

## 6 Conclusion

Biopolymers hold substantial preclinical promise as multifunctional tools in vascularized composite allotransplantation, enabling progress in tissue-specific regeneration, immune modulation, and localized drug delivery. Across the preclinical studies reviewed, both natural and synthetic polymers demonstrated a capacity to enhance vascularization, reduce inflammation, and support structural integration within composite grafts. However, their successful clinical translation remains challenged by material-specific limitations, manufacturing complexity, and the need for tailored approaches based on tissue-specific demands. Insights from SOT, where biopolymers have shown value in controlling immunosuppression, could help inform the next generation of biomaterial strategies in VCA. Going forward, interdisciplinary collaboration among immunologists, biomaterial scientists, pharmacologists, and transplant surgeons is critical to optimizing these technologies. Continued efforts in translational research and standardized evaluation will be essential to unlock the full potential of biopolymers and integrate them into future clinical protocols for reconstructive transplantation.
