# Supplementary material for: Bio-boosting transplants: a systematic review on biopolymers in vascular composite allotransplantation
Source: Front Immunol. 2026 Jan 19;16:1645261. doi: 10.3389/fimmu.2025.1645261 (PMC12861910; doi:10.3389/fimmu.2025.1645261)
Supplement: Supplementary file 1 [file DataSheet1.docx]

**11. SUPPLEMENTARY DIGITAL CONTENT**

**Supplementary digital content 1.** Full search strategy for each database included

| **Database** | **Search String** | **Results** |
| --- | --- | --- |
| PubMed/MEDLINE | ("Tissue Transplantation" OR "vascularized composite allotransplantation"[MeSH Terms] OR "composite tissue allotransplantation" OR "composite tissue transplantation" OR VCA)  AND  ("Biopolymers"[MeSH Terms] OR biopolymer OR biopolymers OR polymer OR polymers OR hydrogel OR hydrogels OR scaffold OR scaffolds OR "natural polymer" OR "synthetic polymer" OR "biodegradable material" OR "biodegradable materials")  Filters applied: Adaptive Clinical Trial, Case Reports, Clinical Study, Clinical Trial, Clinical Trial, Veterinary, Comparative Study, Controlled Clinical Trial, Observational Study, Observational Study, Veterinary, Randomized Controlled Trial, English | 334 |
| Web of Science | (TS=("Tissue Transplantation" OR "Composite Tissue Allotransplantation" OR "Vascularized Composite Allotransplantation" OR "VCA" OR "composite tissue transplantation" OR "vascularized composite tissue"))  AND  (TS=("Biocompatible Materials" OR "Biopolymers" OR "Polymers" OR biopolymer* OR polymer* OR hydrogel* OR scaffold* OR "natural polymer*" OR "synthetic polymer*" OR "biodegradable material*")) | 449 |
| EMBASE | ('composite tissue allotransplantation'/exp OR 'vascularized composite allotransplantation':ab,ti OR 'vca':ab,ti OR 'composite tissue transplantation':ab,ti OR 'vascularized composite tissue':ab,ti)  AND  ('biocompatible material' OR 'biopolymer'/exp OR 'polymer'/exp OR biopolymer*:ab,ti OR polymer*:ab,ti OR hydrogel*:ab,ti OR scaffold*:ab,ti OR 'natural polymer*':ab,ti OR 'synthetic polymer*':ab,ti OR 'biodegradable material*':ab,ti) | 309 |
| Google Scholar | ("vascularized composite allotransplantation" OR "composite tissue allotransplantation" OR "composite tissue transplantation" OR "VCA")  AND  (biopolymer OR biopolymers OR polymer OR polymers OR hydrogel OR hydrogels OR scaffold OR scaffolds OR "natural polymer" OR "synthetic polymer" OR "biodegradable material" OR "biodegradable materials") | 250 |
| Cochrane | *allotransplantation OR composite tissue allotransplantation OR composite tissue transplantation OR VCA in  AND  biopolymer OR biopolymers OR polymer OR polymers OR hydrogel OR hydrogels OR scaffold OR scaffolds OR natural polymer OR synthetic polymer OR biodegradable material OR biodegradable materials | 14 |

**Supplementary digital content 2.** Quality assessment of studies included via the Newcastle-Ottawa Scale scoring system

| **Study (Author, Year)** | **Selection (Max 4)** | **Comparability (Max 2)** | **Outcome (Max 3)** | **Total Score (Max 9)** | **Quality Rating** |
| --- | --- | --- | --- | --- | --- |
| Clark et al. (2018) | 4 | 2 | 3 | 9 | High |
| La Monaca et al. (2018) | 3 | 1 | 2 | 6 | Moderate |
| Abellán et al. (2021) | 4 | 2 | 3 | 9 | High |
| Gallo et al. (2022) | 3 | 2 | 2 | 7 | Moderate |
| Naidu et al. (2023) | 3 | 1 | 2 | 6 | Moderate |

**Supplementary digital content 3.** Quality assessment of studies included via the Level of Evidence scoring system

| **Study (Author, Year)** | **Study Design** | **Level of Evidence** | **Strength of Evidence** |
| --- | --- | --- | --- |
| Clark et al. (2018) | Randomized Controlled Trial | 1b | High |
| La Monaca et al. (2018) | Prospective Cohort Study | 2b | Moderate |
| Abellán et al. (2021) | Randomized Controlled Trial | 1b | High |
| Gallo et al. (2022) | Prospective Cohort Study | 2b | Moderate |
| Naidu et al. (2023) | Randomized Controlled Trial | 1b | High |
| Hornyák et al. (2014) | Experimental Study (In Vivo) | 5 | Foundational Evidence |
| Jank et al. (2015) | Proof-of-Concept (In Vivo) | 5 | Foundational Evidence |
| Duisit et al. (2018) | Preclinical (In Vivo) | 5 | Foundational Evidence |
| Kudryavtseva et al. (2020) | Preclinical (In Vitro/In Vivo) | 5 | Foundational Evidence |
| Sommerfeld et al. (2023) | Preclinical (In Vivo) | 5 | Foundational Evidence |
| Knudsen et al. (2024) | Preclinical (In Vivo) | 5 | Foundational Evidence |

**Supplementary Table 4:** SYRCLE Risk of Bias Tool assessment for Preclinical Studies

| **Study (Author, Year)** | **Selection Bias** | **Performance Bias** | **Detection Bias** | **Attrition Bias** | **Reporting Bias** | **Other Bias** | **Overall Risk** |
| --- | --- | --- | --- | --- | --- | --- | --- |
| Hornyák et al. (2014) | Low | Unclear | Low | Low | Low | Low | Low |
| Jank et al. (2015) | Unclear | High | Unclear | Low | Low | Unclear | Moderate |
| Duisit et al. (2018) | Low | Low | Low | Low | Low | Low | Low |
| Kudryavtseva et al. (2020) | Unclear | Unclear | Unclear | Low | Low | Unclear | Moderate |
| Sommerfeld et al. (2023) | Low | Low | Low | Low | Low | Low | Low |
| Knudsen et al. (2024) | Low | Unclear | Low | Low | Low | Low | Low |
